# Supplementary figures and images for: Enterococcal quorum-controlled protease alters phage infection
Source: bioRxiv. 2024 May 11:2024.05.10.593607. Preprint. [Version 1] doi: 10.1101/2024.05.10.593607 (PMC11100838; doi:10.1101/2024.05.10.593607)

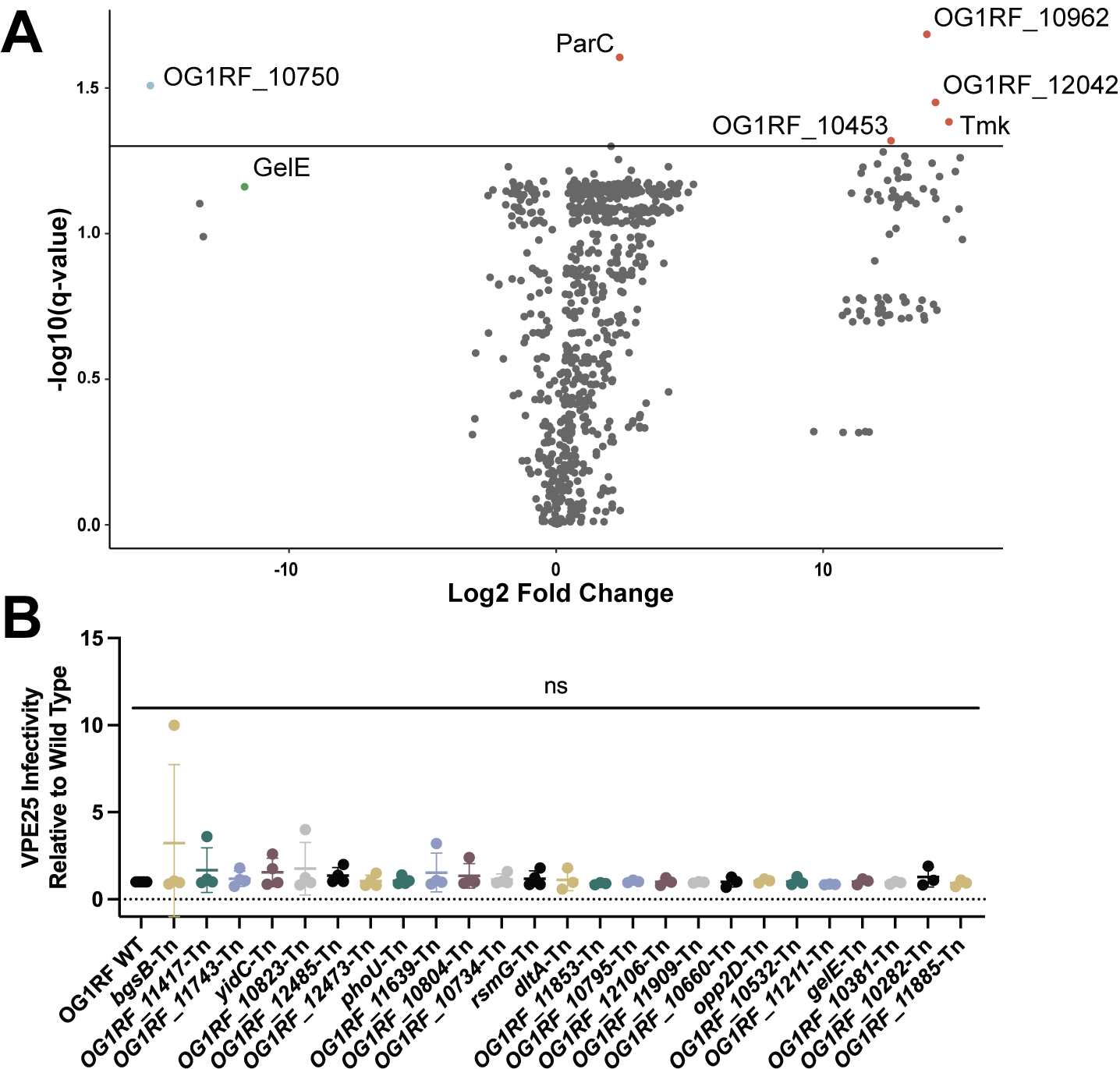

Supplement: Supplement 1 — Supplemental Figure 1: Additional proteomic abundance changes and transposon screen. (A) Volcano plot of bacterial proteins differentially abundant at 20 minutes post-infection between the infected and uninfected samples. Log2-transformed fold change between the two samples is measured on the x-axis. Negative log10-transformed q-value is measured on the y-axis with a line at a significance cut-off of q < 0.05. Points colored in blue are significantly underrepresented in the infected sample when compared to the uninfected sample. Points in red are significantly overrepresented. (B) Transposon mutant screen of phage infection indicates no changes in infectivity relative to OG1RF wild type. Transposon mutants were selected from the most over- or underrepresented proteins at 40 minutes as indicated by the differential abundance ratio (Table 1). Data represents an average of four replicates. Error bars represent standard deviation. Significance determined via one-way ANOVA with correction for multiple comparisons. ns = not significant. [file media-1.tif]

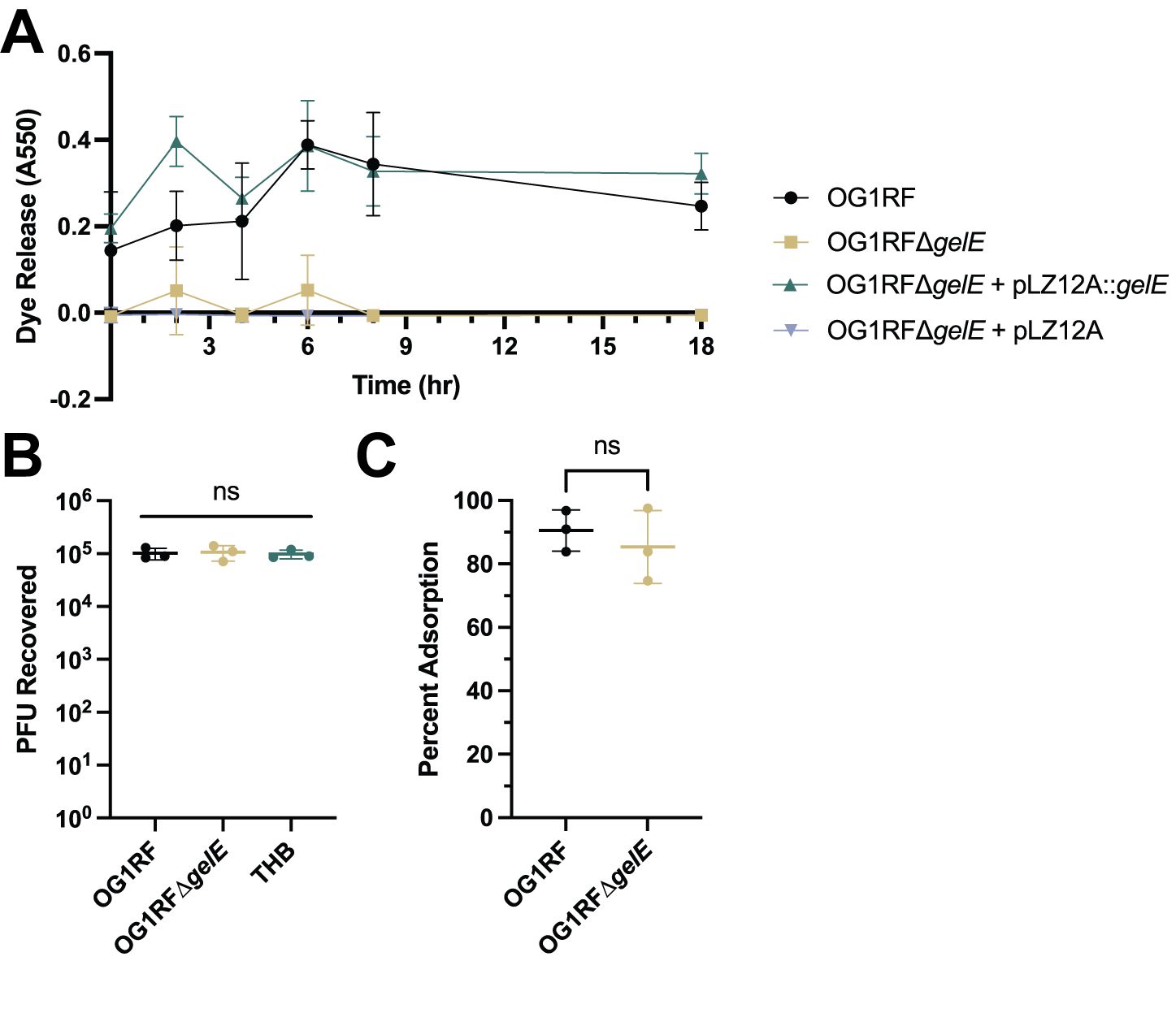

Supplement: Supplement 2 — Supplemental Figure 2: Presence of GelE does not affect virion stability or adsorption. (A) Azocoll cleavage indicates extracellular GelE activity peaks at 6 hours post-subculture in both the wild type strain OG1RF and the complement OG1RFΔgelE::gelE. (B) Treatment of VPE25 virions with spent media has no effect on viable PFUs recovered, regardless of strain. (C) Adsorption of VPE25 to cells is not affected by strain. (B,C) Significance determined using one-way ANOVA with multiple comparisons. ns = not significant. (A, B, C) Data represents an average of three biological replicates. Error bars represent standard deviation. [file media-2.tif]

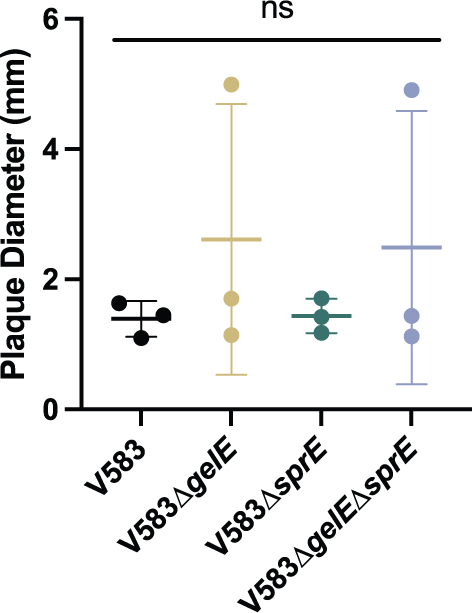

Supplement: Supplement 3 — Supplemental Figure 3: Changes in plaque morphology on V583 are time-dependent. At 24 hours, average VPE25 plaque diameter on V583ΔgelE, V583ΔsprE, and V583ΔgelEΔsprE is not significantly different from wild type. Data represents three biological replicates. Significance determined using one-way ANOVA with multiple comparisons. Error bars represent standard deviation. ns = not significant. [file media-3.tif]

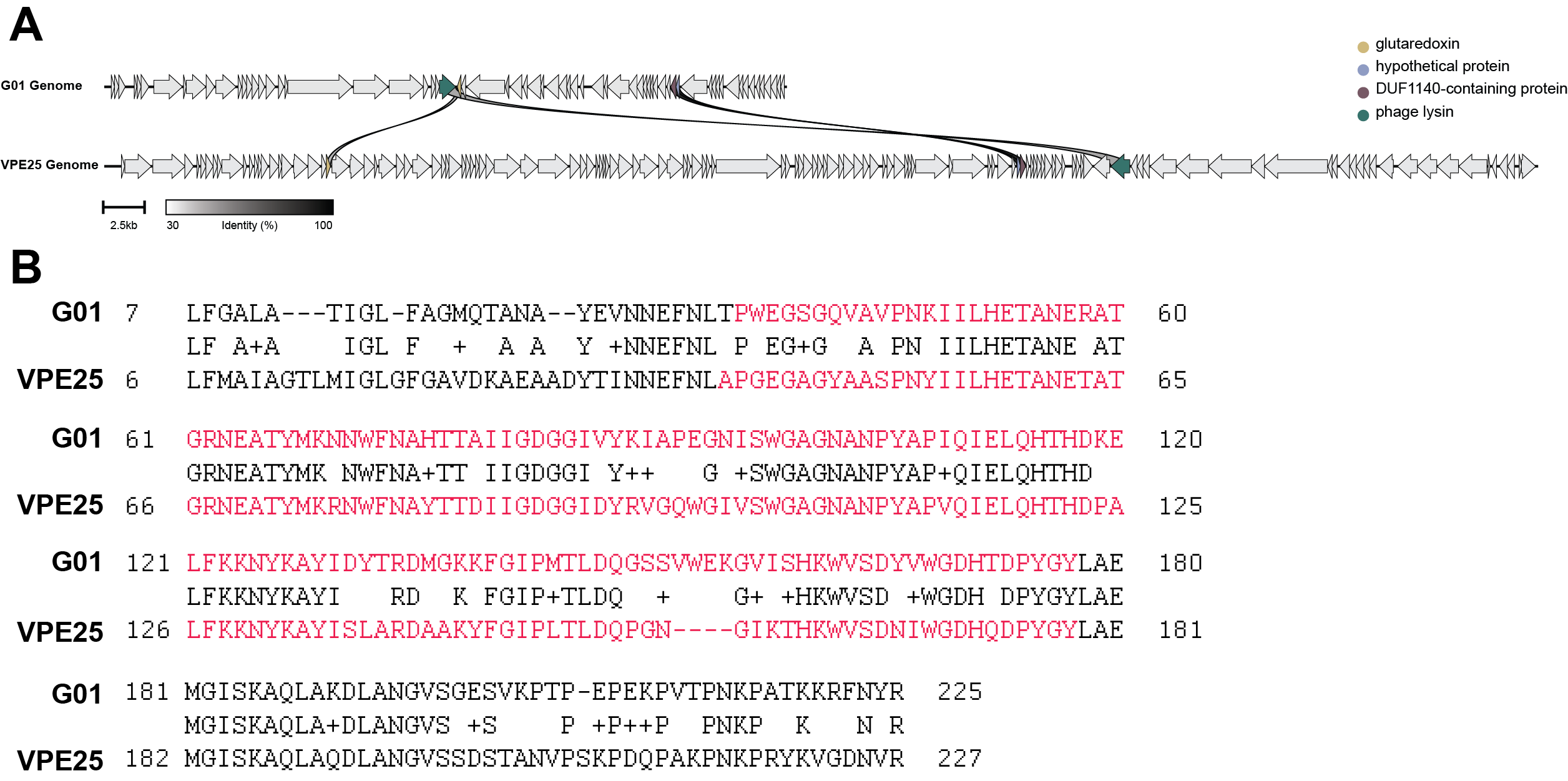

Supplement: Supplement 4 — Supplemental Figure 4: VPE25 and G01 share significant homology in four genes, including their lysins. (A) Comparison of phage genome similarities at the sequence level shows only four genes are conserved with >30% similarity between VPE25 and G01. Figure generated using clinker (81). (B) Schematic of VPE25 and G01 lysin homology generated via Blast 2 sequences (82). Matching amino acids are designated with the corresponding single-letter symbol in the middle lane. Conservative substitutions are marked with a “+”. Red text indicates residues in amidase domain, as identified via InterProScan (73). [file media-4.tif]
